# Supplementary material for: Are carbon emissions trading and green financial instruments synergistic? -Comprehensive quantitative research based on content analysis
Source: PLoS One. 2024 Mar 7;19(3):e0298601. doi: 10.1371/journal.pone.0298601 (PMC10919601; doi:10.1371/journal.pone.0298601)
Supplement: S1 Data — (ZIP) [file pone.0298601.s001.zip › Data/地级市Z_F工作报告.zip!/╣π╢1⁄2/├»├√╩╨/└┤╫╘╣1⁄2╓┌║┼í╛┬φ┐╦╩2╛▌═°í┐_2013.docx]

各位代表：

我代表市人民政府向大会作工作报告，请予审议，并请政协委员和其他列席人员提出意见。

2012年工作回顾

过去一年，在省委、省政府和市委的正确领导下，在市人大及其常委会和市政协的监督支持下，市政府坚持以邓小平理论、“三个代表”重要思想、科学发展观为指导，团结依靠全市人民，狠抓经济社会发展，着力保障和改善民生，圆满完成了十一届人大第一次会议确定的各项任务。全市实现生产总值1951.2亿元，比上年增长10.6%，经济总量在全省东西北地区继续保持领先地位。公共财政预算收入78.1亿元，增长18.2%。全市财政用于民生支出133.6亿元，占支出总额的71.3%。城镇居民人均可支配收入和农村居民人均纯收入实际增长11.9%和16.1%。

一年来，我们主要做了以下工作：

一、着力做大规模总量，经济运行有效加快

去年18个重要经济指标中，我市有10个增速位居全省前六位，其中有5项第一，争先进位的势头越来越明显。

发展动力明显增强。全市完成固定资产投资427.4亿元，增长96.7%，增幅位居全省第一。投资规模在全省的排位比两年前晋升4位。社会消费品零售总额902.2亿元，增长14.8%，增幅居全省第六。进出口总值10.4亿美元，增长12.7%，增幅居全省第五。实际利用外资8005万美元，增长 126.6%，增幅居全省第一。茂名石化炼油改扩建龙头装置开车成功，原油年加工能力达到2000万吨。茂名热电厂“上大压小”扩建工程、10万吨醋酸仲丁酯、15万吨溶剂改质等9个重点项目顺利完工。“反哺工程”扎实推进，全市签订乡贤回归项目332个，计划投资1334.9亿元。

增长结构明显优化。大办工业成效显著，全市规模以上工业增加值增长22.0%，增幅居全省第五；其中地方规模以上工业增加值增长78.6%。全市工业投资增长156.2%，增幅居全省第一。新增规模以上工业企业54家。建成华南地区唯一的国家级危险化学品质检中心。新认定国家级高新技术企业3家、省级民营科技企业17家。专利申请与授权量大幅增长。新增工业类省名牌产品2个。现代农业加快发展，全市农业总产值增长5.1%。新增省农业名牌产品10个、市级以上农业龙头企业14家，农民专业合作社户数位居全省第二。1个农业地方标准获省批准实施，3个省级农业标准化示范区通过验收。全年旅游总收入93.1亿元，增长10.4%。

发展协调性明显改善。市级加大对县一级的激励扶持，去年对六个县（市、区）各项拨款和配套资金达7.87亿元，比上年增长28.5%。县域经济发展速度明显加快，6个县（市、区）实现生产总值1595.18亿元，增长14.9%，占全市总量的81.8%，比上年提高4.8个百分点。县域规模以上工业增加值增长58.1%，投资增长105.3%，公共财政预算收入增长21.8%，均高出全市平均增幅。

二、着力打造发展平台，新区建设有效推进

滨海新区建设取得重大突破。滨海新区正式成立。新区发展总体规划已经省政府审定施行。新区管理架构完成搭建。五大国有开发集团加快组建，茂名港有限公司注册成立。粤电煤炭码头完成投资3亿多元。广州港集团、200万吨特种油项目公司、中化集团等公司的前期项目工作组已进驻我市。新港大道加快施工，将于今年建成。

高新区实现超常规发展。高新区“园镇互动”模式顺利运转，融资难题有效破解。新引进项目31个、总投资41.6亿元，新建成投产项目13个，工业增加值增长42%。一批投资超10亿元的大项目加快推进，世界化工龙头企业德国巴斯夫公司落户高新区。

各地工业园区建设加快推进。茂南石化工业园产业集聚势头良好，新建成一批规模较大的项目。茂港中润工业园挂牌运作，现有意向入园项目16个。信宜产业转移园新引进项目10个，规模以上工业总产值增长138.5%。高州新增规模以上工业企业19家，金山工业园新征土地500亩。化州杨梅工业园完成首期70%的市政工程，民营科技工业园在建和建成企业15家。电白产业转移工业园落户企业达78家，总产值增长76.2%。

三、着力加强基础建设，发展条件有效优化

交通建设持续加快。累计投入18.29亿元，新、改建公路695公里（包括县道以下公路）。高水公路高州段路面改造全面完工，茂化公路改扩建项目开工建设。环城东路二期和海洋大道东段加快施工，海洋大道西段和油城十路前期工作已完成。包茂高速公路茂名段前期工作基本完成，征地拆迁即将开展。茂湛铁路建设快速推进。深圳至茂名铁路茂名段、汕湛高速公路茂名段、博贺新港区疏港铁路前期工作加紧推进。湛江机场迁建选址工作进一步优化。茂名港全年吞吐量达到2390万吨。博贺新港区规划完成调整，茂名港总体规划编制取得实质性进展。

市政设施不断完善。官山五路、西粤南路建成通车，站前五至七路以及文光路改造工程全面完成，官渡四路建设加快推进。市区新增绿地9.5万平方米，绿化覆盖率达42.3%。城乡清洁工程全面开展。生活垃圾焚烧发电项目基本建成。高州垃圾无害化处理场垃圾填埋区投入使用，化州垃圾处理场垃圾填埋区基本建成，电白、信宜垃圾处理场正加快施工。

民生水利建设全面铺开。全年完成水利投资16.69亿元，水利保障经济社会发展能力明显增强。农村饮水安全工程全面完成，新解决71.5万人饮水不安全问题。高州水库灌区改造工程顺利推进，已完成123公里渠道改造。

资源环境和生态建设工作扎实有效。节能减排约束性指标全面下降。高州水库水源和生态环境保护取得新成效。组建市土地开发储备中心，全年新增建设用地指标1.15万亩，较好地保障了重点建设项目的用地需求。森林覆盖率达57.38%，省政府批准我市为“广东省林业生态市”。

四、着力推进改革创新，发展活力有效增强

深入推进行政体制改革。行政服务中心建设不断加强，平均每月业务量超过1.13万宗，行政审批效率明显提高。6个县（市、区）行政服务中心全部建成运作。大力推进镇（街）行政服务中心建设。启动新一轮行政审批制度改革，将大幅度清理压减审批事项。市公共资源交易中心挂牌运作，公共资源交易监管体制改革加快推进。完成茂南、茂港两区大部门体制改革，全面启动县级大部门体制改革，深化简政强镇事权改革。完成全市事业单位分类改革。

加快推进经济体制改革。市国资委正式挂牌运作。制定下发镇级财政管理体制改革指导意见，下一步将在全市全面实施。金融生态环境进一步优化，金融改革发展活力明显增强。汇丰银行茂名支行正式开业，实现小额贷款公司县域全覆盖，3家企业进入上市程序，政策性涉农保险工作有效开展。全市人民币贷款余额增长21.6%，增速位居全省第三。

五、着力加强公共服务，人民生活有效改善

就业和社会保障成绩显著。企业职工养老保险全年净增参保人数20.2万人，新增人数超过前10年的总和，参保率提高到63.5%，在全省的排名迅速上升五位，工作经验在全省推广。实现新农保和城镇居民养老保险全覆盖。城镇居民医保和新农合实现并轨，参保率达98.6%。全市镇级人力资源和社会保障服务所全部开工建设，2/3已完工。城镇新增就业6.31万人，新增转移就业12.86 万人，城镇登记失业率为2.75%。新开工建设保障性住房2978套，有1261套完工或接近完工。城乡低保救助和农村五保供养标准进一步提高。“扶贫济困日”募捐善款5305万元。建立低收入群众生活补贴双联动机制，向市直低收入群众发放生活补贴358.8万元。累计建成平价商店286家，总数居全省第三。

教育强市工作加快推进。已有10个镇向省申报教育强镇。122所外出务工人员子弟学校、268所义务教育规范化学校、25所乡镇中心幼儿园和83所村级幼儿园建设任务如期完成，学前三年毛入园率、义务教育规范化学校覆盖率、高中阶段教育毛入学率分别达85.5%、53.15%和89.6%。农村义务教育债务有效化解。校安工程全面完成。全市统一公选校长、重奖名师和市直学校整合、高中均衡招生等改革顺利实施。农村教师全面享受“特岗”津贴。

平安建设成效显著。“三打两建”工作有效推进，重点地区整治成效明显，全市刑事发案、特别是重大案件大幅下降，人民群众安全感不断增强。综治信访维稳工作有新进步。安全生产形势稳定向好，交通、火灾事故四项指标全面下降。

各项社会事业取得新成绩。公共文化服务体系不断完善，文化惠民成效明显，茂名大讲堂等文化活动有效开展，完成国有文艺院团改制工作。基层医疗卫生机构运行新机制基本建立，区域医疗卫生信息化项目有序推进。通过省卫生城市复审。高度重视和加强人口计生工作，全力稳定低生育水平。体育、国防动员、民兵预备役、拥军优抚安置、食品药品、民族宗教、妇女儿童、慈善、残疾人事业、统计、审计、法律援助、应急、人防、气象、地震、殡改、档案、驻外机构、地方志、红十字等工作取得新的成绩。

同时，政府自身建设不断加强。自觉接受市人大及其常委会和市政协的监督，共办理人大代表建议84件、政协委员提案250件。加强行政复议和行政应诉工作，落实行政执法责任制。加强行政监察、审计监督和政务公开。积极开展“加快转型升级，建设幸福茂名”重点项目监督检查，开展专项效能监察，加大问责力度，全年共问责121人。加大民主评议政风行风和纠风工作力度，政府执行力明显提升。

去年政府工作报告决定的110项工作，有104项按期完成，有6项正在稳步推进。

过去一年的工作，成绩来之不易。这是在省委、省政府和市委的正确领导下，全市人民齐心协力、奋力拼搏的结果。在此，我代表市政府向全市人民，向驻茂人民解放军、武警官兵和各民主党派、人民团体、社会各界人士致以崇高敬意！向所有关心支持茂名发展的港澳同胞、台湾同胞、海外侨胞及国际友人，表示衷心感谢！

尽管去年我市经济社会发展取得了显著成绩，但总体发展水平不高的问题还相当突出，主要体现在经济总量偏小、人均水平偏低，人均GDP只有全国平均水平的85.9%、全省平均水平的60.9%。对外交通明显落后，城镇化水平明显偏低，民生领域仍存在不少薄弱环节。我们要敢于正视这些困难和问题，并采取强有力措施逐步加以解决。

2013年工作安排

今年是全面贯彻落实党的十八大精神的开局之年，做好今年的工作意义十分重大。我们要坚持以中国特色社会主义理论为指导，全面贯彻落实胡春华书记视察茂名重要讲话精神，牢固树立发展是硬道理的思想，围绕人均生产总值力争2015年底超过全国平均水平的发展目标，采取切实可行的措施，全力加快发展。在理念上，要不甘落后、敢于争先，不等不靠、奋发图强，以坚定的信心和决心，大干快干实干，奋力摆脱落后面貌。在举措上，要以加强交通建设为突破口，以滨海新区建设为重大平台，以发展临海现代产业为主攻方向，以加快城镇化发展为抓手，以惠民利民为落脚点，着力促进经济社会加快发展、协调发展。在方法上，要紧盯目标、开拓创新抓落实，把目标要求细化到一个个具体工作中，苦干巧干，敢想会干，讲一件、做一件、成一件。

今年的主要预期目标是：生产总值增长12%，固定资产投资增长30%，公共财政预算收入增长12%，城镇居民人均可支配收入增长11%，农村居民人均纯收入增长12%，居民消费价格涨幅控制在3.4%左右，单位生产总值能耗下降3.89%。

重点要抓好以下八项工作：

一、兴交通，强基础，全力争创区域发展新优势

突出发展对外交通。千方百计加快交通建设，加强与珠三角和周边省区市联系，改变我市发展的区位劣势。今年交通建设计划投资36亿元，比去年翻一番，新改建公路800公里(包括县道以下公路)。一是加快对外交通快捷化建设。年内建成茂湛铁路茂名段。力争上半年开工建设包茂高速茂名段、年内开工建设深茂铁路江门至茂名段。分步实施油城十路及包茂高速茂名东连接线建设。加快推进湛江机场迁建、汕湛高速公路茂名段、茂名综合客运枢纽、西粤南路立交工程前期工作。二是加快港口建设。加快推进茂名港总体规划的修改完善和报批工作，争取年内获得批复。支持粤电煤码头及航道加快建设，争取明年竣工。年内开工建设博贺新港区防波堤工程。加快推进广州港集团通用码头4个泊位的前期工作，争取年内开工建设。

大力推进市县区基础设施一体化建设。尽快建成以市区为中心、带动200多万人口的半小时生活圈，以交通的快速连接，带动人流、物流、信息流的密切流动。加快推进茂化公路改扩建，全力争取尽快完成。抓紧启动国道207线信宜至高州段路面改造工程。加快各地城区与镇之间的道路建设。

加强水利建设。抓好民生水利五项重点工作，全年水利建设计划投资20.3亿元。加快高州水库灌区节水改造，大力推进省级水利示范县、小型农田水利重点县、中小河流治理、中小型灌区改造、海堤达标加固、鉴江高岭拦河闸等项目建设，加快推进滨海新区供水、高州水库恢复89米蓄水位、高州水库灌区节水改造二期等工程的立项和建设。

此外，还要抓好电力、通讯等基础设施建设，全年电网建设计划投资4.32亿元。

二、抓新区，促起步，全力打造转型发展新平台

抓好基础性工作。明确滨海新区管委会的具体权责和运行机制。加快组建新区五大国有开发集团。创新用人机制，在全国范围内招聘新区急需的各类人才。抓紧完成新区城市总体规划的报批，加快推进土地利用总体规划修编、控制性详细规划编制以及规划环评工作。加快新区路网建设，年内建成新港大道，年初完成环城东路二期工程，上半年完成海洋大道东段和西城西路建设。争取年内完成博贺新港区疏港铁路前期报批工作。

抓好起步区建设。划出一定范围作为滨海新区起步区，出台起步区管理暂行办法，集中力量，加快建设，以起步区开发建设带动新区的大发展。理顺管理体制，增强新区统筹发展的能力。积极引进战略投资者，引导各类企业参与起步区开发建设。

大力发展临海产业。做足海的“文章”，大力发展临海现代产业。加快推进博贺国家级中心渔港建设。重点推进中化茂名储运项目、30万吨天然纤维素一体化项目、博贺2×100万千瓦火电、200万吨特种油等石化、能源项目。着眼长远，围绕港口优势，着手谋划一批临海工业重大项目。

三、强招商，兴产业，全力开创加快发展新局面

努力提高招商实效。整合招商力量，打造招商平台，精心办好各类招商活动。运用各地茂名商会的力量，深入开展“反哺工程”。实施招商引资奖励办法，充分引导社会力量，扩大招商引资。重点推进临海产业、农产品深加工和矿产资源招商，吸引产业集聚。加强部门协作，加大项目跟踪协调力度，促使新引进项目顺利落地建设。

大力发展工业。一是加快完成炼油厂、乙烯厂卫生防护距离居民搬迁安置工作，支持茂名石化发展壮大。加强地企合作平台建设，合作建设一批优质石化中下游项目。推进质量强市。积极发挥国家危险化学品质检中心(广东)的作用。二是强化园镇融合发展，加强重大项目和高素质人才的谋划引进，加快高新区发展步伐。加强部门协作，大力推进园区征地工作。组建高新区开发集团，完善园区投融资体系。年内开工建设巴斯夫项目，全年力争15个项目新开工，20个项目建成投产。三是壮大县域工业。出台县(市、区)工业园区建设考核办法，安排专项资金支持县级工业园区建设，鼓励先进。全年力争各县(市、区)工业园区新增工业投资、工业增加值增速在25%以上，各园区新开工项目不少于5个。扶持发展镇级工业，继续开展“工业十强镇”评选表彰活动。全年力争增加规模以上工业企业40家。

推进重点项目建设。抓好重大项目的谋划储备、立项报批和开工建设，以大项目带动大发展。瞄准央企、省属国企、行业领先企业，引进落实一批优质项目。做好项目前期工作，着力解决用地难、融资难、项目落地难等问题。今年安排重点项目110项，年度计划投资184.5亿元。

大力发展现代服务业。加强政银企合作，推动银行、保险、证券业加快发展。鼓励建立多元化金融机构，创新金融产品和服务方式，开展农村金融改革创新综合试验，大力支持企业上市融资，进一步改善金融生态环境。整合滨海旅游和山区生态旅游资源，加强整体策划宣传，打造特色旅游品牌，加快发展滨海旅游和生态旅游。推动物流、信息等产业加快发展，大力建设商业联合体。

四、扩容量，提质量，全力加快城市发展新步伐

加快区域中心城市建设。坚持向东向南靠海发展战略，拉开城市发展框架，启动环水东湾新城重点区域建设，引导人口、资源等要素向新区聚集，一心一意把城市做大做强，增强中心城区的辐射带动力。

大力实施城市升级行动计划。抓好道路优化，两年内完成对市区主要道路的沥青罩面改造，实现道路“黑底化”，加快建设市区东北片小区路网，实施市区西出口改造。抓好节能亮化和临街美化，逐步对市区路灯进行LED改造。抓好城市绿化，加强交通管理和城市管理，全面整治乱摆乱卖现象。做好露天矿区整体移交和管理工作。提高市民素质，推进人的城镇化。

强化用地保障。加强土地开发储备工作，搞活土地市场。优先保证重点项目建设用地。加强与农垦系统合作，共同开发建设用地。加强土地交易监管，保障公开竞争、公平交易。

建设宜居城乡。大力实施城乡清洁工程，深入开展宜居城乡创建活动。全面实施生活垃圾处理费征收方式改革。抓好节能减排和环境保护工作，推进垃圾无害化处理设施和污水处理厂建设。启动高州水库至名湖水库全封闭供水工程前期工作。大力支持生态功能区建设，推进森林进城围城等生态景观工程建设，努力建设美丽茂名。加快水东湾综合整治、高州水库生态保护和环境治理工作。

五、重三农，强县域，全力构建城乡统筹发展新格局

大力改善农村生产生活条件。在努力壮大县域工业的同时，进一步加大对县域的扶持力度，着力统筹城乡发展。鼓励推进农村综合改革。加强农村公共服务建设，积极推进农村公益事业“一事一议”财政奖补工作。稳步实施创建幸福村居五年行动计划，加快名镇名村示范村建设步伐。扎实推进新一轮扶贫开发工作。

大力发展现代农业。提高农业产业化发展水平，扶持壮大农产品深加工企业和农业龙头企业。深入实施“一村一品、一镇一产业”战略和“放心食品名牌工程”，抓好现代特色农业基地和农业标准化示范区建设。建设特色鲜明、功能配套的农产品加工和现代物流园区。加快农业品牌培育、发展和推广。积极拓展农产品市场。

切实增加农民收入。采取综合措施，营造有利环境条件，拓宽增收渠道，确保农民收入与城镇居民收入同步增长。加强农民专业合作组织和农村集体经济组织建设，带动农民发展生产，增加收入。依法维护好农民合法权益。

加快县城和中心镇建设。加强城镇基础设施和公共服务设施建设，完善城市功能，加速城市人口和产业集聚。鼓励各县(市)中心城区大力提高绿化水平，改善提升城市环境。加强中心镇建设，增强中心镇辐射带动力。

积极壮大民营经济。认真落实鼓励民营经济发展的各项政策措施。加快社会信用体系和市场监管体系建设，创造良好的营商环境。强化服务，积极帮助民营企业排忧解难。大力扶持，做大做强一批工业骨干企业。加强对民营企业家的培训，组织企业家到先进地区培训学习。加强产学研对接，促进民营经济发展上水平。

六、促改革，勇突破，全力增强创新发展新活力

深化行政管理体制改革。市级要在今年第一季度、县级要在今年上半年基本完成本轮行政审批制度改革，大幅度压减行政审批、备案、核准事项。继续推进县级大部制改革。深化事业单位分类改革，有序推进行政执法体系改革。加快构建覆盖市、县、镇三级的综合政务服务体系，完善市、县行政服务中心建设，年内全面建成镇（街）行政服务中心。全面推进电子政务建设，强化各级行政服务中心的电子政务服务管理，上半年建成市网上办事大厅，年底前建成各县（市、区）网上办事大厅，实现各级网上办事大厅互联互通。年内启动“茂名通”政府服务热线及电子政务平台。完善公共资源交易改革体制，加强对公共资源交易的监管。

深化经济领域各项改革。大力培育创税型项目，加强税收征管，促进财政收入稳定增长，提高财政收入质量。加快推行镇级财政体制改革，今年市财政对按照时间进度实施试点的镇（街），各补助20万元，连续补助2年。加强国有资产监管制度建设，加快推进国有资产整合重组，提高国有经济运营质量和效率。深化探矿权、采矿权有偿使用制度改革，培育矿业权市场。

大力提升对内对外开放水平。壮大出口产业，培育拳头出口产品，扶持重点出口企业，充分利用各类出口平台，扩大外贸出口。完善利用外资工作机制，切实提高利用外资工作水平。认真落实与珠海市合作框架协议。

七、惠民生，强服务，全力营造和谐发展新态势

实施充分就业和全民社保行动计划。社会保障是人民群众生活安康、家庭幸福的重要基础，我们要落实政策，逐步完善社会保障体系。一是抓好就业工作。全面落实就业扶持政策，解决重点人群就业问题。开展“百镇百村农村劳动力技能提升培训工程”和“百区千户城镇就业援助工程”，促进农村劳动力转移就业和城镇困难群体就业。提高企业最低工资标准，加强劳动监察。全年实现城镇新增就业6万人以上。二是抓好社会保险工作。巩固企业职工养老保险扩面成果，力争全市参保率达到70%以上。完善城乡医保一体化运作机制，城乡居民医保财政补助标准提高到每人每年330元，住院支付比例平均提高10个百分点，符合条件的困难弱势群体由政府全额资助参保。城乡居民养老保险月基础养老金从55元提高到65元，企业退休人员基本养老金增长10%左右。提高社保经办能力，建立“一站式结算”服务模式。加快完成镇(街)人力资源和社会保障服务平台建设。三是抓好住房保障工作。完善住房保障体系，启动市区第九期保障性住房建设。四是抓好困难群众救助工作。落实好孤残儿童基本生活保障标准，建立孤残儿童基本生活保障标准自然增长机制。提高五保供养标准和补助水平。加强低保工作，确保城乡低收入困难家庭全部纳入低保。

大力发展科教文卫事业。大力推进教育创强工作，促进城乡教育均衡、优质发展。义务教育全面纳入公共财政保障范围，优先保障教育财政支出。创建36个教育强镇，茂港建成教育强区。加快规范化幼儿园和义务教育规范化学校创建步伐，义务教育规范化学校覆盖率达到57%。努力抓好高中阶段教育，完善职业教育体系，支持高等教育发展。大力推进教育信息化建设。加强教师队伍建设，坚持在全市公选中小学校长，充实农村学校师资力量。完善关系教师切身利益的绩效工资、社保制度，对山区、边远农村义务教育教师发放津补贴。提高城乡免费义务教育生均公用经费补助标准。加强学校安全管理。深化医药卫生体制改革，切实解决群众“看病难、看病贵”问题。提升基层医疗服务水平，加快村卫生站规范建设。今年县级以下基层医疗卫生机构医师要增加250人，增长10%；全市每万人医疗床位数量力争达到全省平均水平。全面推进区域医疗卫生信息化建设。加强对医疗服务收费和药品价格的监督检查。全面落实国家基本药物集中网上采购制度和药物遴选制度。加快推进县级公立医院综合改革。大力开展科技创新工作，加强创新平台建设，积极推进产学研合作。努力繁荣文化事业，积极发展文化产业，扎实推进公共文化服务体系建设，加快推进市文化中心、青少年活动中心项目前期工作。

大力发展各项社会事业。积极发展养老服务事业，推进颐年苑养老基地建设，加强社区养老服务设施建设，年内全市增加2000个养老床位。以茂南区为试点，创新发展社区居家养老服务业。加强人口计生工作，稳定低生育水平，全面实现“创优”目标。发展体育事业，广泛开展群众体育活动。做好青年、妇女儿童、残疾人工作。完善低收入群众生活补贴双联动机制；深入推进平价商店建设，年内新建农副产品平价商店30家、平价药店30家。加强国防建设，做好拥军优抚工作。认真组织实施第三次全国经济普查。提高殡葬公共服务水平。做好外事、侨务、对台、民族宗教、统计、打私、打假、知识产权、地震、气象、人防、档案、方志、慈善和老区等工作。

加强和创新社会管理。扎实推进“平安茂名”建设，建立立体网络化的社会治安打防管控体系，完善科技强警设施建设，严密防范和依法打击各类违法犯罪活动，全力保障经济发展和人民群众生命财产安全。完善流动人口管理机制。深入推进“三打两建”工作，营造良好社会经济环境。加强安全生产监管，强化公共安全体系和企业安全生产基础建设，遏制重特大安全事故。完善食品药品网上监管平台，提高食品药品安全管理水平。认真落实农村基层组织工作经费保障。加强社会矛盾化解，落实信访维稳工作责任。健全应急管理体系，提高突发事件应对处置能力。强化工会、共青团、妇联等群团组织的枢纽功能，培育发展和规范管理社会组织。强化网络虚拟社会建设管理。

八、重实干，求实效，全力强化务实发展新作风

加强民主法制建设。认真落实人大及其常委会各项决议决定，坚持向人大及其常委会报告工作，支持人民政协履行职能。认真办理人大代表建议和政协委员提案，提高办理质量。坚持科学民主决策，完善决策程序。就经济社会发展重大问题广泛协商，虚心听取各民主党派、人民团体和社会各界意见。坚持依法行政，进一步落实依法行政各项制度。加强审计和监察工作。推进反腐倡廉建设，规范权力运行，确保政府清廉。加大政府信息公开力度。自觉接受新闻舆论监督，充分发挥网络的监督作用。

全力以赴抓落实。要以真抓实干的作风，推动各项任务有效落实。一要锤炼好的作风。严格执行中央《八项规定》，进一步改进文风会风和调查研究的方式方法，做到重实际、重实干、重实效，短会、短言、短时间落实。领导干部要带头深入基层，带头深入工作一线，靠前指挥，协调解决具体问题，推动工作顺利开展。大力弘扬艰苦奋斗、勤俭节约的优良作风。认真开展以为民务实清廉为主要内容的党的群众路线教育实践活动。二要建立好的机制。完善督查机制，加大督查力度，逐一督促落实政府工作报告确定的各项任务。完善对县（市、区）的考核，启动对市直部门的考核，鼓励勇于承担责任，推动政府工作全面落实。市直部门要善于从基层实践中发现和总结行之有效的工作模式，树立榜样，以点带面推广实施。三要打造好的队伍。严格监督管理，加强培养锻炼，努力建设一支敢于干事、勇于开拓、善于执行的干部队伍。

各位代表，展望新的一年，我们满怀信心！让我们团结一致，在市委的正确领导下，全面贯彻落实党的十八大精神，解放思想，锐意进取，奋力开创茂名更加美好的未来！
